# Carcinosarcoma of the ampulla of Vater: a case report and literature review

**DOI:** 10.1186/s40792-016-0233-7

**Published:** 2016-09-27

**Authors:** Hideki Izumi, Naoki Yazawa, Daisuke Furukawa, Yoshihito Masuoka, Misuzu Yamada, Taro Mashiko, Yohei Kawashima, Masami Ogawa, Yoshiaki Kawaguchi, Tetsuya Mine, Kenichi Hirabayashi, Toshio Nakagohri

**Affiliations:** 1 Department of Gastrointestinal Surgery, Tokai University School of Medicine, 143 Shimokasuya, Isehara, Kanagawa 259-1193 Japan; 2 Department of Internal Medicine, Tokai University School of Medicine, 143 Shimokasuya, Isehara, Kanagawa 259-1193 Japan; 3 Department of Pathology, Tokai University School of Medicine, 143 Shimokasuya, Isehara, Kanagawa 259-1193 Japan

**Keywords:** Carcinosarcoma, Ampulla of Vater, Pancreaticoduodenectomy

## Abstract

**Background:**

Carcinosarcoma of the ampulla of Vater is extremely rare, and to the best of our knowledge, this is the third reported study.

**Case presentation:**

The patient was a 73-year-old man, who presented with a chief complaint of dark urine. After a work-up, we suspected duodenal papillary cancer and performed a subtotal stomach-preserving pancreaticoduodenectomy with lymph node dissection. Immunohistochemically, the sarcomatous atypical cells were diffusely positive for cytokeratin AE1&3 and vimentin and focally positive for α-smooth muscle actin; these cells were also negative for desmin, CD34, DOG1, c-kit, and S100. From these findings, we diagnosed the patient with so-called carcinosarcoma. There was no lymph node metastasis.

**Conclusions:**

Carcinosarcoma of the ampulla of Vater has a poor prognosis, and lymph node metastases are often seen. For the complete cure of carcinosarcoma of the ampulla of Vater, resection with the dissection of the lymph nodes may be necessary.

## Background

Carcinosarcomas are rare malignant tumors that are composed of both carcinomatous and sarcomatous elements that grow intermingled with each other [1]. This tumor type has been detected in many different organs, such as the uterus, lung, hepatobiliary tract, gastrointestinal tract, and urinary tract [2–5]. However, carcinosarcoma of the ampulla of Vater is extremely rare. To the best of our knowledge, only two cases of carcinosarcoma of the ampulla of Vater have been reported in the English literature [6–8]. In this report, we present a case of carcinosarcoma of the ampulla of Vater.

## Case presentation

A 73-year-old man had presented to his local hospital with a chief complaint of dark urine. He underwent an endoscopic retrograde biliary drainage procedure and was subsequently referred to our hospital with a diagnosis of obstructive jaundice caused by tumors of the duodenal papilla. He had no past medical or family history. During the first visit, his blood tests were normal because his jaundice had been reduced by the previous physician. In the tumor marker testing, we saw only a mild elevation in the carcinoembryonic antigen (5.8 ng/mL).

**Fig. 1:**
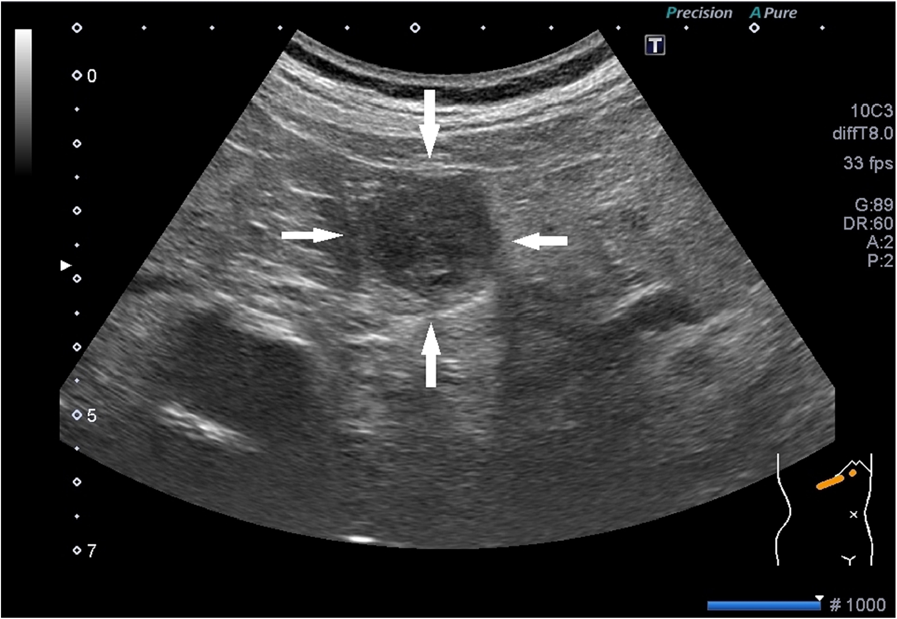
A non-homogenous low echoic tumor mass was identified (*white arrow*)

**Fig. 2:**
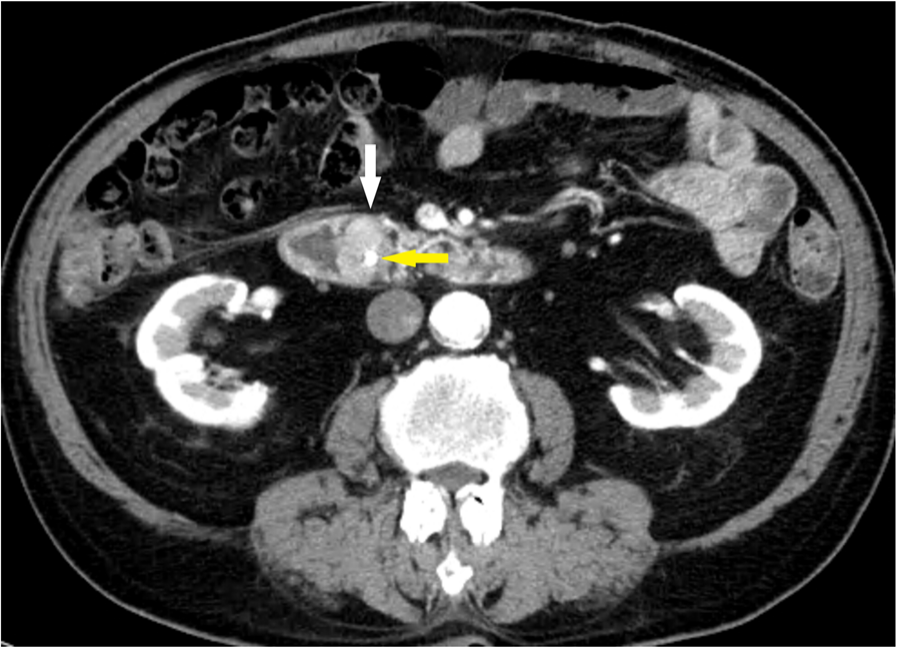
A mass with mild contrast effects was identified (*white arrow*). The *yellow arrow* indicates endoscopic retrograde biliary drainage (ERBD)

**Fig. 3:**
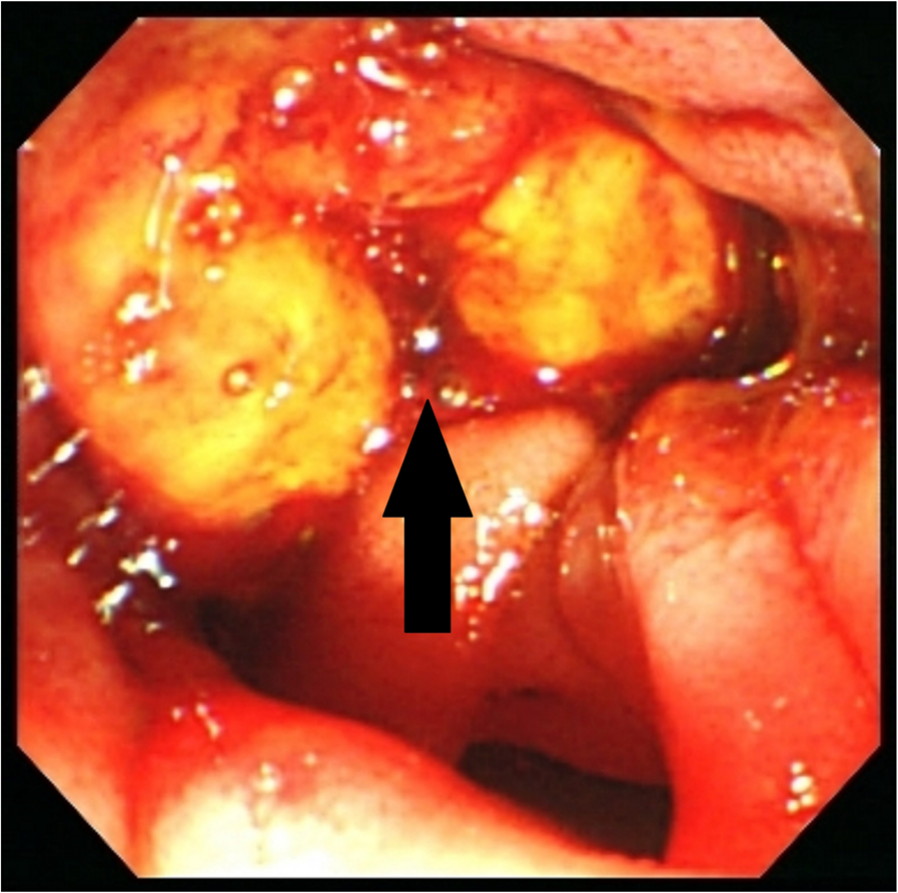
The figure depicts a type 2-like lesion that was centered on the papilla. The *black arrow* indicates the ampulla of Vater

Using abdominal ultrasound (Fig. 1), we found a boundary ambiguity that corresponded to the papilla of Vater, and a non-homogenous low echoic tumor mass. Using computed tomography scan with contrast (Fig. 2), we identified a mass with mild contrast effects that also corresponded to the papilla of Vater. On endoscopic analysis (Fig. 3), hemorrhagic type 2-like lesion with central ulcer that was located around the papilla was seen; the biopsy was positive for poorly differentiated adenocarcinoma. Based on these findings, we diagnosed the patient with duodenal papillary lesion poorly differentiated glands cancer; we performed a subtotal stomach-preserving pancreaticoduodenectomy with a lymph node dissection.

**Fig. 4:**
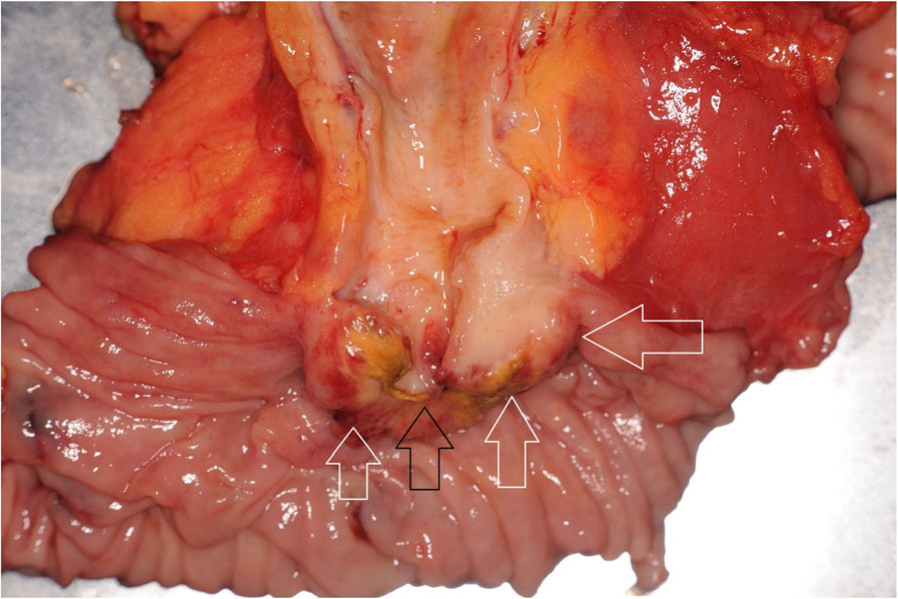
A whitish and solid tumor was identified in the ampulla of Vater. The *black arrow* indicates the ampulla of Vater and the *white arrow* indicates the tumor

**Fig. 5:**
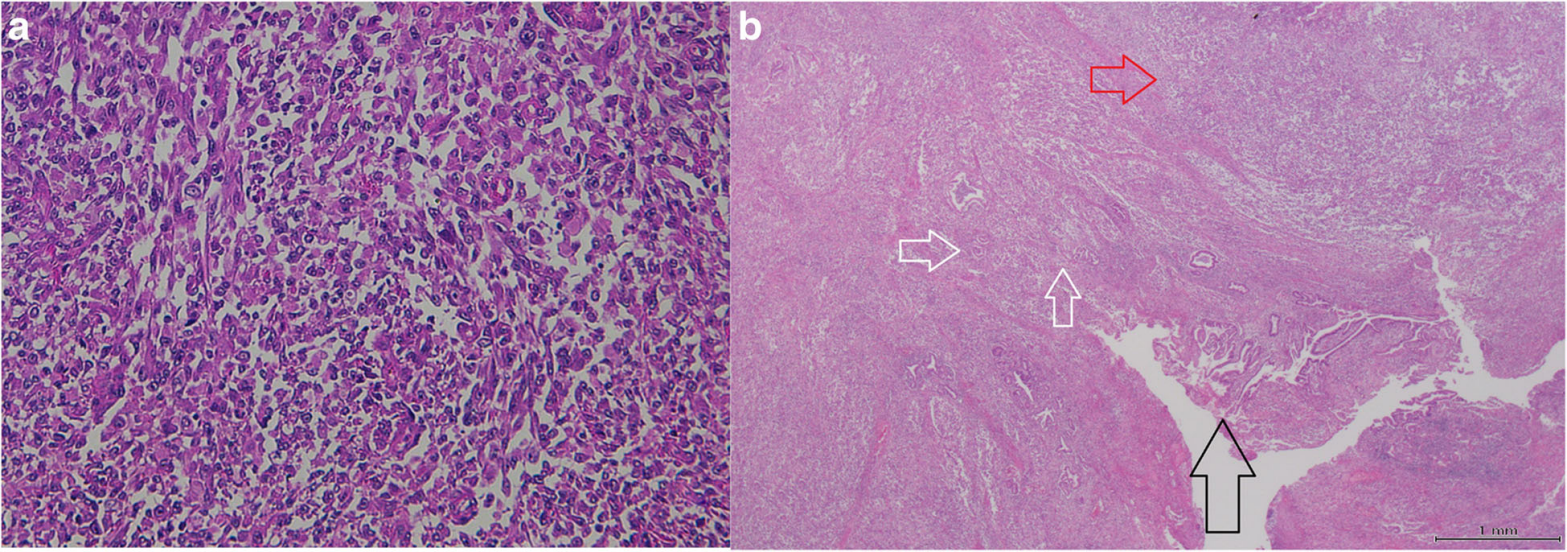
**a** The sarcomatous tissue composed of large and pleomorphic spindle cells. **b** The tumor of the ampullary lesion (*black arrow*) consisted of two divergent components: adenocarcinoma (*white arrows*) and sarcomatous tissue (*red arrow*)

**Fig. 6:**
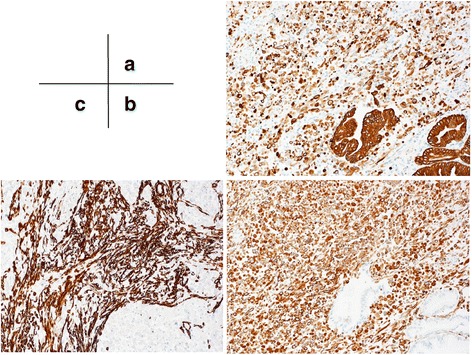
**a** The cytokeratin AE1&3 and **b** vimentin stains were positive and **c** α-smooth muscle actin

Macroscopically, the well-demarcated, whitish, solid tumor was identified in the ampulla of Vater. The tumor was 20 × 10 mm in diameter (Fig. 4). Microscopically, most of the tumor was composed of diffuse sarcomatous atypical cells with spindle-like or epithelioid-like features (Fig. 5a). These sarcomatous atypical cells had irregular-shaped, pleomorphic nuclei with prominent nucleoli. There was no specific tissue differentiation in the tumor such as osseous, muscular, or cartilaginous. The irregular-shaped tubular structures were the foci of the adenocarcinoma and were focally intermingled with the sarcomatous atypical cells. We observed a transition between the adenocarcinoma cells and sarcomatous atypical cells (Fig. 5b). Immunohistochemically, the sarcomatous atypical cells were diffusely positive for cytokeratin AE1&3 (Fig. 6a) and vimentin (Fig. 6b), focally positive for α-smooth muscle actin (Fig. 6c) and negative for desmin, CD34, DOG1, c-kit, and S100. From these findings, we diagnosed this tumor as a so-called carcinosarcoma. The patient had no lymph node metastases.

The postoperative course was uneventful, and the patient was discharged on the 16th postoperative day. Since discharge, the patient has received no adjuvant treatment and remains free of recurrent disease after 5 months of follow-up.

### Discussion

In this case, we identified two important clinical issues. Carcinosarcoma of the ampulla of Vater is extremely rare. To obtain a complete cure for this type of tumor, an en bloc resection with the dissection of the lymph nodes will be necessary.

First, the carcinosarcoma of the ampulla of Vater is extremely rare. To the best of our knowledge, this is the third description of a case of carcinosarcoma of the ampulla of Vater (Table 1). In the biliary tract, carcinosarcoma of the gallbladder is the most common with fewer than 70 cases described [9, 10]. Several cases of intrahepatic cholangiocarcinoma with sarcomatoid changes and extrahepatic cholangiocarcinoma with sarcomatous features have been reported in the literature. Although the esophagus is the most frequent location for carcinosarcoma [11], carcinosarcomas have been detected in many different organs, such as the uterus, lung, hepatobiliary tract, gastrointestinal tract, and urinary tract [2–5].

**Table 1:** Three reported cases of surgical resection of carcinosarcoma of the Ampulla of Vater

Author	Year	Sex	Age	Chief complaint	Pathological findings	Operation	Lymph node metastasis	Prognosis
Kench [6]	1997	F	46	Melena, lassitude, dyspnea, hypotension	Adenocarcinoma cells, squamous carcinoma cells and spindle-type sarcoma cells (so-called)	PD	N+	Died (POD 240 due to liver metastasis)
Kijima [7]	1999	M	46	Jaundice, liver dysfunction	Adenocarcinoma cells and spindle-type sarcoma cells (so-called)	PD	Unknown	Unknown
Present case	2015	M	73	Jaundice	Adenocarcinoma cells and spindle-type sarcoma cells (so-called)	PD	N−	Alive (POM 5)

Second, to achieve a complete cure for carcinosarcoma of the ampulla of Vater, the patient needs to undergo an en bloc resection with the dissection of the lymph nodes. The published cases on carcinosarcoma of the ampulla of Vater have been summarized in Table 1. Lymph node metastases were found in one of the three cases. For other types of carcinosarcomas, such as primary lung carcinosarcoma, lymph node metastasis was observed in half of them [12]. Carcinosarcoma is a disease with a high rate of lymph node metastasis and appears to be required for lymph node dissection in resection. The article with prognostic mention of the patient was one such case; this patient died of recurrent liver metastases 8 months after the surgery. This form of carcinosarcoma appears to have a poor prognosis. In other organs, the prognosis for carcinosarcoma is very poor, and many of these patients have died within 1 year after surgery from liver metastases [12–14]. According to Okabayashi et al. [14], the overall 1-, 2-, and 3-year survival rate in carcinosarcoma of the gallbladder after surgery, which is the most common site of cancer in the biliary tract, were 37.2, 31.0, and 31.0 %. However, some cases [15] may achieve long-term survival after resection, and aggressive resection should be performed with curative intent where possible. Surgery is suggested as the only recognized treatment for carcinosarcomas; radiotherapy and chemotherapy have no benefit on survival [16]. Several targets, such as EGFR, c-kit, and Cox-2, have been identified [17]; therefore, establishment of chemotherapy for carcinosarcomas is extremely difficult.

Carcinosarcoma is a tumor that has epithelial components (cancer) and non-epithelial components (sarcoma) intermingled into one lesion [18]. For the sarcomatous components in carcinosarcoma, various tissue images are generally observed from the one which indicates differentiation to bone, cartilage, muscle, or fat. Those that do not indicate clear mesenchymal differentiation are classified as “true carcinosarcomas”; these show differentiations into specific interstitial tissues in the sarcoma portion of the tumor such as rhabdomyosarcoma, leiomyosarcoma, chondrosarcoma, osteosarcoma, and “so-called carcinosarcoma.” In these lesions, the sarcoma-like tissue is mainly composed of spindle-shaped cells but does not differentiate into the clear interstitial tissue [19]. True carcinosarcoma has three features. The first is the presence of a genuine sarcomatous component including chondrosarcoma, osteosarcoma, rhabdomyosarcoma, and leiomyosarcoma. The second is that there is no transitional zone between the carcinomatous and sarcomatous components. The third is that the sarcomatous component is positive for mesenchymal markers and negative for epithelial markers. However, it remains controversial as to whether the lesion must satisfy all the above histological features to be diagnosed as true carcinosarcoma. The tumor in this case had for a large portion of sarcomatous components that was based on spindle shape cell proliferation and cytokeratin. The tumor was positive for vimentin and was diagnosed as a so-called carcinoma. Because the epithelial components and non-epithelial components are intermingled, chemotherapy has not been successful in cases of metastasis and relapse. The development of an appropriate chemotherapy regimen is essential to improve the prognosis of carcinosarcoma.

## Conclusions

We have presented an extremely rare case of carcinosarcoma of the ampulla of Vater. Similar to other cancers like sarcoma, duodenal papillary cancer is a disease with poor prognosis; however, pancreaticoduodenectomy involving the dissection of the lymph nodes appeared to be necessary for a complete cure. To improve prognosis, a better chemotherapy regimen is mandatory.

## Consent

Written informed consent was obtained from the patient for the publication of this case report and all accompanying images. A copy of the written consent form is available for review for the Editor-in-Chief of this journal.

## Abbreviations

ERBD: Endoscopic retrograde biliary drainage
